# Association of COVID-19 Pandemic with Colorectal Cancer Screening: Impact of Race/Ethnicity and Social Vulnerability

**DOI:** 10.1245/s10434-024-15029-x

**Published:** 2024-02-15

**Authors:** Muhammad Muntazir Mehdi Khan, Muhammad Musaab Munir, Selamawit Woldesenbet, Yutaka Endo, Mujtaba Khalil, Diamantis Tsilimigras, Alan Harzman, Emily Huang, Matthew Kalady, Timothy M. Pawlik

**Affiliations:** 1 Division of Surgical Oncology, Department of Surgery, The Ohio State University Wexner Medical Center and James Comprehensive Cancer Center, Columbus, OH USA; 2 Division of Colorectal Surgery, Department of Surgery, The Ohio State University Wexner Medical Center and James Comprehensive Cancer Center, Columbus, OH USA

**Keywords:** Cancer screening, Colorectal cancer, COVID-19 pandemic

## Abstract

**Background:**

The COVID-19 pandemic disrupted health care delivery, including cancer screening practices. This study sought to determine the impact of the COVID-19 pandemic lockdown on colorectal cancer (CRC) screening relative to social vulnerability.

**Methods:**

Using the Medicare Standard Analytic File, individuals 65 years old or older who were eligible for guideline-concordant CRC screening between 2019 and 2021 were identified. These data were merged with the Center for Disease Control Social Vulnerability Index (SVI) dataset. Changes in county-level monthly screening volumes relative to the start of the COVID-19 pandemic (March 2020) and easing of restrictions (March 2021) were assessed relative to SVI.

**Results:**

Among 10,503,180 individuals continuously enrolled in Medicare with no prior diagnosis of CRC, 1,362,457 (12.97%) underwent CRC screening between 2019 and 2021. With the COVID-19 pandemic, CRC screening decreased markedly across the United States (median monthly screening: pre-pandemic [*n* = 76,444] vs pandemic era [*n* = 60,826]; median Δ*n* = 15,618; *p* < 0.001). The 1-year post-pandemic overall CRC screening utilization generally rebounded to pre-COVID-19 levels (monthly median screening volumes: pandemic era [*n* = 60,826] vs post-pandemic [*n* = 74,170]; median Δ*n* = 13,344; *p* < 0.001). Individuals residing in counties with the highest SVI experienced a larger decline in CRC screening odds than individuals residing in low-SVI counties (reference, low SVI: pre-pandemic high SVI [OR, 0.85] vs pandemic high SVI [OR, 0.81] vs post-pandemic high SVI [OR, 0.85]; all *p* < 0.001).

**Conclusions:**

The COVID-19 pandemic was associated with a decrease in CRC screening volumes. Patients who resided in high social vulnerability areas experienced the greatest pandemic-related decline.

**Supplementary Information:**

The online version contains supplementary material available at 10.1245/s10434-024-15029-x.

Colorectal cancer (CRC) is the third most common cancer among both men and women in the United States.^1,2^ Since 1970, screening for CRC has been used as an effective method to detect precancerous polyps and early-stage cancer.^3,4^ According to the most up-to-date cancer screening guidelines, the United States Preventive Services Task Force (USPSTF) recommends screening for all adults ages 45 to 74 years.^5^ As a result, CRC incidence has notably declined by approximately 1% per year during the past decade due to the earlier diagnosis and treatment of pre-cancerous polyps during screening colonoscopies performed for this age cohort.^1^

In the United States, COVID-19 has been diagnosed for nearly 1 billion individuals since the declaration of a global pandemic by the World Health Organization (WHO) in March 2020.^6^ To mitigate transmission of the virus, various policies with an impact on outpatient care were implemented such as conversion of routine office visits into telehealth appointments and cancellation of non-urgent interventions such as screening and vaccination.^7^ These policies affected primary health care by redirecting resources to help with the management of the pandemic.^8,9^ Notably, cancer screening recommendations also were altered. The American Society for Gastrointestinal Endoscopy (ASGE) recommended the delay of routine screening colonoscopies and follow-up procedures after positive stool tests.^10^

Despite removal of COVID-19 restrictions, the decline in rates of screening for cancers such as breast and lung tumors have not fully recovered to date.^10,11^ The rate of recovery in screening deficits was notably delayed among the Medicaid or Medicare-Medicaid dual-eligible populations compared with privately insured patients.^12^

Potential changes in the diagnosis and management of CRC after the COVID-19 pandemic have not been well-defined. Possible changes include delays in treatment initiation and an increased incidence of advanced disease at the time of presentation.^13–15^ Previous studies have demonstrated inequalities in cancer screening among certain racial/ethnic groups and socioeconomically vulnerable populations.^16,17^ Moreover, the risk of CRC increases by age.^18^ Siegel et al.^2^ estimated that more than 56% of CRCs in 2023 would be diagnosed for individuals age 65 years or older.^2^ As such, delays in cancer screening due to the COVID-19 pandemic among older patients and a return to pre-pandemic levels after lockdown restrictions are eased may have important implications.^11,19^ Therefore, the current study aimed to characterize how the COVID-19 pandemic influenced CRC screening. In addition, the study sought to determine the impact of the COVID-19 pandemic lockdown on CRC screening relative to social vulnerability among a nationally representative cohort of older individuals.

## Methods

### Data Source and Study Population

Data were extracted from 100% Medicare Standard Analytic Files claims data obtained by the Centers for Medicare and Medicaid Services (CMS) between 2019 through 2021 to identify individuals who did and did not undergo screening for CRC.^20^ The CMS maintains the Medicare Standard Analytic Files (SAF), an administrative billing database that provides patient-level data on demographics, diagnoses, procedures, and expenditures.^21^ To be included in the analytic cohort, individuals had to have Medicare coverage and consecutive enrollment in Medicare Parts A and B for the study duration.

Only individuals age 65 years or older were included in the study cohort. The International Classification of Diseases, tenth revision (ICD-10) diagnostic and procedure codes, Current Procedural Terminology (CPT) codes, and Healthcare Common Procedure Coding System (HCPCS) codes were used to identify beneficiaries who had undergone screening for CRC.^22,23^ These codes included various methods for CRC screening including colonoscopies, barium enema, and fecal-occult blood test (Appendix Table S1).^24^

Individuals with a prior diagnosis of CRC, as identified by ICD codes, were excluded from the study cohort. This study was approved by The Ohio State University Institutional Review Board, and the need for informed patient consent was waived because the data were de-identified.

### Study and Outcome Measures

Information on age, sex, race/ethnicity, geographic region, Social Vulnerability Index (SVI), and Charlson Comorbidity Index (CCI) were collected.^20,25^ According to the U.S. census, regions were defined as Northeast (New England, Middle Atlantic), South (South Atlantic, East South Central, and West South Central), Midwest (East North Central, West North Central), and West (Mountain, Pacific).^26^

The SVI, developed and maintained by the Center for Disease Control and Agency for Toxic Substances and Disease Registry, is a validated measure used to assess community vulnerability to external pressures.^27^ The SVI was linked with the Medicare Standard Analytical Files using county-level Federal Information Processing System (FIPS) codes and categorized into tertiles.^27^

The participants were categorized into three equal groups based on tertile values for SVI. The CCI was assessed using ICD-9-CM and ICD-10-CM codes and dichotomized as ≤2 versus >2.^20,25^ Race/ethnicity was recorded based on self-reported social constructs, which are not a reflection of genetic ancestry, and categorized as non-Hispanic White, non-Hispanic Black, Hispanic, and non-Hispanic other race/ethnicity.^28^ The non-Hispanic other race/ethnicity group was categorized as American Indians, Alaskan Natives, Native Hawaiians, Asians, Pacific Islanders, and self-reported “other” race/ethnicity groups due to small sample sizes.^28^

The primary outcome of interest was monthly median screening volumes for each time period. Monthly volumes of CRC screening were recorded for the entire study duration. The study duration was divided into three periods. The pre-COVID-19 pandemic period was defined as January 2019 to February 2020, whereas the COVID-19 pandemic period was defined as March 2020 to March 2021, and the post-pandemic period was defined as April 2021 to December 2021.^29^

Lockdown restrictions were eased throughout the country at different times and in a stepwise fashion. However, the majority of states had eased COVID-related restrictions by March 2021, so this was defined as the cutoff for the post-COVID-19 era^30,31^

### Statistical Analyses

Continuous variables were analyzed via the Wilcoxon rank-sum test and reported as median and interquartile range (IQR). Categorical variables were compared via the chi-square test and represented as frequencies. Multivariable logistic regression models were used to assess the odds of a patient undergoing screening for CRC. Models for screening were adjusted for sex, race, age, CCI, SVI, genetic susceptibility, region, index year, and rurality (non-metropolitan vs metropolitan). The ICD codes were used to identify individuals with genetic susceptibility for malignancy (Appendix Table S2).^22^ Statistical analyses were performed using STATA version 18, and a two-sided significance level of α equal to 0.05 was used.

## Results

### Demographic Characteristics

Among 10,503,180 beneficiaries age 65 years or older who were continuously enrolled in Medicare between 2019 and 2021, 1,362,457 (12.97%) were screened for CRC. Overall, the median age was 69 years (interquartile range [IQR], 67–72 years) and approximately one half of the cohort was female (*n* = 5,679,955, 54.1%). The majority of the individuals (*n* = 8,781,793, 83.6%) had a CCI score of 2 or lower. In terms of race/ethnicity, most of the individuals were non-Hispanic White (*n* = 9,179,865, 87.4%), whereas a smaller proportion of individuals were non-Hispanic Black (*n* = 574,883, 5.5%), Hispanic (68,461, 0.7%), or non-Hispanic other (*n* = 679,971, 6.5%).

Most of the individuals resided in metropolitan areas (*n* = 8,349,172, 79.5%) in the southern (*n* = 4,245,230, 40.4%) or the midwestern (*n* = 2,281,677, 21.7%) region of the United States. Notably, the individuals from high versus low SVI counties were more likely to be non-Hispanic Black (9.4% vs 2.6%) or Hispanic (1.2% vs 0.3%) and to reside in metropolitan areas (80.6% vs 77.8%) in the southern (55.4% vs 25.0%) or western (25.8% vs 13.5%) regions of the United States (all *p* < 0.001; Table 1).

**Table 1:** Characteristics of study cohort by Social Vulnerability Index (SVI)

Variable	Total *n* (%)	Low SVI *n* (%)	Moderate SVI *n* (%)	High SVI *n* (%)	*p* Value^a^
Age (years)					
Mean	69.60 ± 3.05	69.58 ± 3.05	69.61 ± 3.05	69.62 ± 3.05	<0.001
Median (IQR)	69 (67–72)	69 (67–72)	69 (67–72)	69 (67–72)	
Race^b^					
White	9,179,865 (87.4)	3,205,106 (91)	3,072,574 (88.2)	2,902,185 (82.9)	<0.001
Black	574,883 (5.5)	92,191 (2.6)	154,617 (4.4)	328,075 (9.4)	
Hispanic	68,461 (0.7)	9669 (0.3)	17,969 ( 0.5)	40,823 (1.2)	
Other	679,971 (6.5)	214,710 (6.1)	236,748 (6.8)	228,513 (6.5)	
Sex					
Male	4,823,225 (45.9)	1,622,711 (46.1)	1,600,577 (46)	1,599,937 (45.7)	<0.001
Female	5,679,955 (54.1)	1,898,965 (53.9)	1,881,331 (54)	1,899,659 (54.3)	
Metro					
Metro	8,349,172 (79.5)	2,738,200 (77.8)	2,789,470 (80.1)	2,821,502 (80.6)	<0.001
Non-metro	2,154,008 (20.5)	783,476 (22.2)	692,438 (19.9)	678,094 (19.4)	
Region					
Midwest	2,281,677 (21.7)	1,194,335 (34)	724,259 (20.8)	363,083 (10.4)	<0.001
Northeast	1,842,438 (17.5)	967,136 (27.5)	578,785 (16.6)	296,517 (8.5)	
South	4,245,230 (40.4)	880,642 (25)	1,426,436 (41)	1,938,152 (55.4)	
West	2,129,247 (20.3)	475,573 (13.5)	751,934 (21.6)	901,740 (25.8)	
Missing	4588	3990	494	104	
CCI					
≤2	8,781,793 (83.6)	2,939,415 (83.5)	2,911,348 (83.6)	2,931,030 (83.8)	<0.001
>2	1,721,387 (16.4)	582,261 (16.5)	570,560 (16.4)	568,566 (16.2)	
Genetic susceptibility					
No	10,491,331 (99.9)	3,517,072 (99.9)	3,478,085 (99.9)	3,496,174 (99.9)	<0.001
Yes	11,849 (0.1)	4604 (0.1)	3823 (0.1)	3422 (0.1)	

IQR, interquartile range; Metro, metropolitan area; Non-metro, non-metropolitan area; CCI, Charlson Comorbidity Index

^a^Statistically significant: *p* < 0.05

^b^Race is reported as non-Hispanic White, non-Hispanic Black, Hispanic, non-Hispanic other.

### Association of the COVID-19 Pandemic CRC Screening Volumes with SVI

**Fig. 1:**
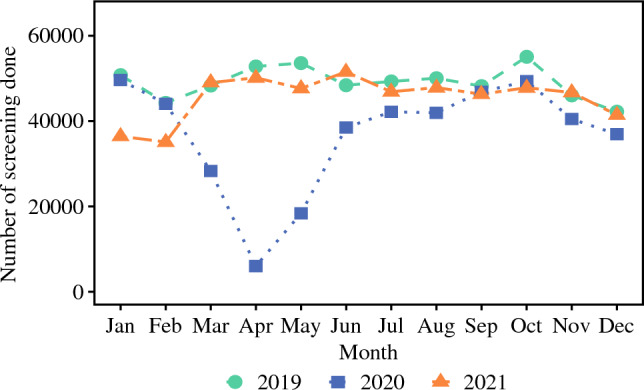
Overall monthly median screening volumes before, during, and after the COVID-19 pandemic.

**Fig. 2:**
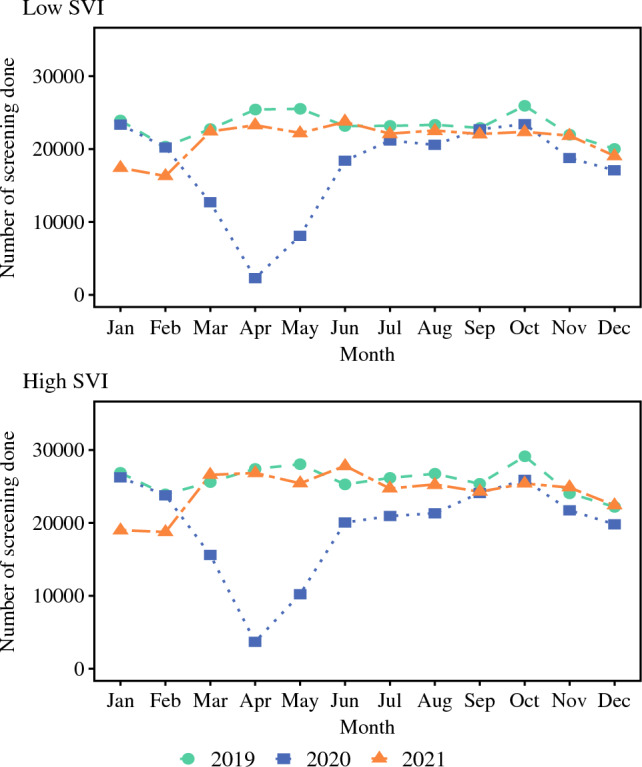
Monthly median screening volumes for low and high SVI categories before, during, and after the COVID-19 pandemic.

The monthly median volumes of CRC screening decreased markedly after the start of the pandemic (*n* = 60,826) versus before the pandemic (*n* = 76,444) (*p* < 0.001; Fig. 1). Compared with the pre-pandemic period, the monthly median CRC screening volumes during the pandemic were lower across all SVI categories as follows: low (*n* = 18,402 vs 23,136; median Δ*n* = 4734 [%Δ 20.46%]), moderate (*n* = 22,224 vs 27,375; median Δ*n* = 5151 [%Δ 18.78%]), high (*n* = 20,064 vs 25,859; median Δ*n* = 5795 [%Δ 22.41%]) (Fig. 2). Moreover, the screening volumes were markedly lower during the COVID-19 pandemic among the male (*n* = 27,655 vs 33,960; median Δ*n* = 6305 [%Δ 18.57%]) and female (*n* = 33,171 vs 42,400; median Δ*n* = 9229 [%Δ 21.77%]) patients, as well as among the non-Hispanic White (*n* = 54,255 vs 67,242; median Δ*n* = 12,987 [%Δ 19.31%]), non-Hispanic Black (*n* = 3390 vs 4121; median Δ*n* = 731 [%Δ 17.74%]), Hispanic (*n* = 335 vs 456; median Δ*n* = 121 [%Δ 26.54%]), and non-Hispanic other (*n* = 1600 vs 2157; median Δ*n* = 557 [%Δ 25.82%]) individuals, (all *p* < 0.001).

In the post-pandemic period, after March 2021, the monthly median CRC screening volumes generally rebounded to pre-pandemic levels (*n* = 74,170) and were much higher than during the COVID pandemic in 2020 (*n* = 60,826) (*p* < 0.001). Notably, during the post-pandemic period versus during the pandemic in 2020, the monthly median CRC screening volumes rose among all SVI categories as follows: low SVI (*n* = 22,200 vs 18,402; median Δ*n* = 3798 [%Δ 20.64%]), moderate SVI (*n* = 26,262 vs 22,224; median Δ*n* = 4,038 [%Δ 18.17%]), high SVI (*n* = 25,258 vs 20,064; median Δ*n* = 5,194 [%Δ 25.89%]) (*p* < 0.001).The median CRC screening volumes also rose among non-Hispanic White (*n* = 64,708 vs 54,255; median Δ*n* = 10,453 [%Δ 19.27%]) non-Hispanic Black (*n* = 4156 vs 3390; median Δ*n* = 766 [%Δ 22.60]), Hispanic (*n* = 444 vs 335; median Δ*n* = 109 [%Δ 32.54%]), and non-Hispanic other (*n* = 2192 vs 1600; median Δ*n* = 592 [%Δ 37.00%]) individuals (all *p* < 0.001) (Table 2).

**Table 2:** Median monthly CRC screening volumes before, during, and after the COVID-19 pandemic

Characteristics	Pre-COVID	During COVID	Post-COVID	*p* Value^a^
	Jan 2019 to Feb 2020	Mar 2020 to Mar 2021	Apr 2021 to Dec 2021	
Overall	76,444	60,826	74,170	<0.001
Race^b^				
White	67,242	54,255	64,708	<0.001
Black	4121	3390	4156	<0.001
Hispanic	456	335	444	<0.001
Others	2157	1600	2192	<0.001
Sex				
Female	42,400	33,171	40,744	<0.001
Male	33,960	27,655	32,793	<0.001
SVI				
Low	23,136	18,402	22,200	<0.001
Moderate	27,375	22,224	26,262	<0.001
High	25,859	20,064	25,258	<0.001
Metropolitan				
Metro	52,939	41,107	51,424	<0.001
Nonmetro	23,223	18,392	22,058	<0.001
Region				
Midwest	23,331	18,902	22,425	<0.001
South	24,386	20,172	22,656	<0.001
West	14,883	12,318	14,637	0.002
Northeast	13,586	10,123	13,431	0.007

CRC, colorectal cancer; SVI, Social Vulnerability Index; Metro, metropolitan area; Non-metro, Non-metropolitan area

^a^Statistically significant: *p* < 0.05

^b^Race is reported as non-Hispanic White, non-Hispanic Black, Hispanic, non-Hispanic other.

**Fig. 3:**
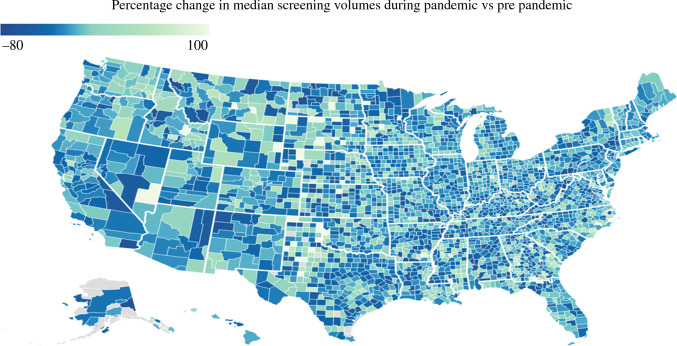
Map showing changes in colorectal cancer (CRC) monthly median screening volumes during the pandemic versus pre-pandemic.

**Fig. 4:**
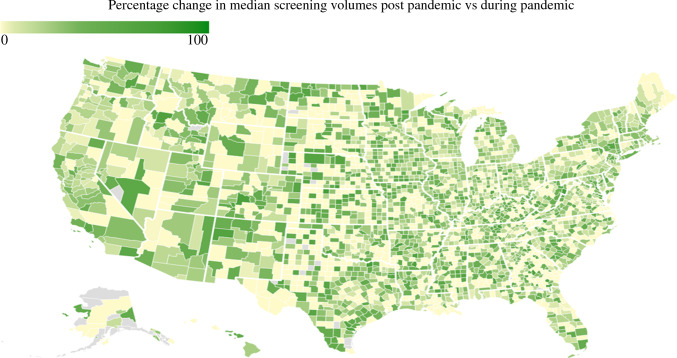
Map showing changes in colorectal cancer (CRC) monthly median screening volumes post-pandemic versus during the pandemic.

Interestingly, there was marked geographic variation in the decline, as well as an increase in CRC screening volumes across the United States (Figs. 3 and 4). Notably, the Northeastern region had the greatest decline in CRC screening (*n* = 10,123 vs 13,586; median Δ*n* = 3,436 [%Δ 25.29%]) and experienced a marked increase (*n* = 13,431 vs 10,123; median Δ*n* = 3308 [%Δ 32.68%]) after the easing of lockdown restrictions. In contrast, the Southern region of the United States experienced the least fluctuation in CRC screening volumes during the COVID-19 pandemic: decrease (*n* = 20,172 vs 24,386; median Δ*n* = 4214 [%Δ 17.28%]); increase (*n* = 22,656 vs 20,172; median Δ*n* = 2484 [%Δ 12.31%]).

In the multivariable logistic regression analysis, residence in high SVI counties was associated with lower odds of screening for CRC, especially during the pandemic era (pre-pandemic [high SVI: OR, 0.85; 95% CI, 0.84–0.85], pandemic era [high SVI: OR, 0.81; 95% CI, 0.81–0.82], post-pandemic [high SVI: OR 0.85; 95% CI, 0.85–0.86]; *p* < 0.001). Similarly, Hispanic and patients who self-identified as “other race” also were associated with lower odds of screening across all periods, with the lowest odds noted during the pandemic era (reference [non-Hispanic White]; pre-pandemic [Hispanic: OR, 0.88; 95% CI, 0.85–0.91; other race: OR, 0.86; 95% CI, 0.85–0.88]; pandemic era [Hispanic: OR, 0.75; 95% CI, 0.54–0.82; other race: OR, 0.79; 95% CI, 0.78-0.81]; post-pandemic [Hispanic: OR, 0.78; 95% CI, 0.74–0.82; other race: OR, 0.89; 95% CI, 0.87–0.91]; *p* < 0.001). In contrast, patients who self-identified as Black were associated with increased odds of screening for CRC throughout the study period. (reference [non-Hispanic White]; pre-pandemic [non-Hispanic Black: OR, 1.04; 95% CI, 1.02–1.05]; pandemic era [non-Hispanic Black: OR, 1.06; 95% CI, 1.04–1.07]; post-pandemic [non-Hispanic Black: OR, 1.07; 95% CI, 1.06–1.09]; *p* < 0.001) (Table 3).

**Table 3:** Multivariable logistic regression analysis of patients undergoing CRC screening

	Pre-COVID	*p* Value	During COVID	*p* Value	Post-COVID	*p* Value^a^
Characteristics	(Jan 2019 to Feb 2020)		(Mar 2020 to Dec 2020)		Jan 2021 to Dec 2021	
Age	0.98 (0.98–0.98)	<0.001	0.97 (0.96–0.97)	<0.001	0.96 (0.95–0.96)	<0.001
Race^b^						
White	Ref.		Ref.		Ref.	
Black	1.04 (1.02–1.05)	<0.001	1.06 (1.04–1.07)	<0.001	1.07 (1.06–1.09)	<0.001
Hispanic	0.88 (0.85–0.91)	<0.001	0.79 (0.75–0.82)	<0.001	0.78 (0.74–0.82)	<0.001
Other	0.86 (0.85–0.88)	<0.001	0.79 (0.78–0.81)	<0.001	0.89 (0.87–0.91)	<0.001
Sex						
Male	Ref.		Ref.		Ref.	
Female	1.03 (1.02–1.03)	<0.001	1.00 (1.00–1.01)	0.4171	1.03 (1.02–1.04)	<0.001
SVI						
Low	Ref.		Ref.		Ref.	
Moderate	0.92 (0.91–0.92)	<0.001	0.92 (0.92–0.93)	<0.001	0.92 (0.91–0.93)	<0.001
High	0.85 (0.84–0.85)	<0.001	0.81 (0.81–0.82)	<0.001	0.85 (0.85–0.86)	<0.001
Region						
West	Ref.		Ref.		Ref.	
Midwest	1.43 (1.42–1.44)	<0.001	1.39 (1.38–1.41)	<0.001	1.44 (1.43–1.46)	<0.001
Northeast	1.05 (1.04–1.05)	<0.001	0.98 (0.97–0.99)	0.002	1.07 (1.05–1.08)	<0.001
South	0.90 (0.90–0.91)	<0.001	0.88 (0.87–0.89)	<0.001	0.87 (0.86–0.88)	<0.001
CCI						
≤2	Ref.		Ref.		Ref.	
>2	1.46 (1.45–1.47)	<0.001	1.54 (1.52–1.55)	<0.001	1.47 (1.45–1.48)	<0.001
Genetic susceptibility						
No	Ref.		Ref.		Ref.	
Yes	2.17 (2.06–2.29)	<0.001	2.22 (2.08–2.37)	<0.001	2.16 (2.01–2.33)	<0.001

CRC, colorectal cancer; SVI, Social Vulnerability Index; Metro, metropolitan area; Non-metro, non-metropolitan area; CCI, Charlson Comorbidity Index

^a^Statistically significant: *p* < 0.05

^b^Race is reported as non-Hispanic White, non-Hispanic Black, Hispanic, non-Hispanic other.

## Discussion

According to the American Cancer Society, there would be 106,970 new cases of colon cancer and 46,050 new cases of rectal cancer in the United States in 2023.^32^ Prior studies have demonstrated that earlier diagnosis of CRC is associated with a significantly higher 5-year survival (> 90%).^32^ Screening for CRC may serve as a highly effective and efficient tool to identify pre-cancerous or early CRC.^33^ Various methods for screening CRC have been introduced in the past few decades, including colonoscopy, sigmoidoscopy, double-contrast barium enema, computed tomographic colonography, fecal occult blood test, and fecal DNA tests.^33–35^ The declaration of a global pandemic by the World Health Organization (WHO) in March 2020 led to a drastic decrease in health care delivery and screening worldwide.^36–40^ Although previous research has assessed the impact of the COVID-19 pandemic on screening for other cancers, the effect of lockdown restrictions on CRC screening volumes, especially among vulnerable populations, remains ill-defined.^13,36^ The current study was important because we specifically characterized the decline in CRC screening during the pandemic, as well as the subsequent recovery in screening volumes relative to county-level SVI and race/ethnicity in a nationally representative cohort. Notably, median monthly CRC screening volumes declined drastically in 2020 after the implementation of lockdown restrictions, with socially vulnerable populations affected the most. Patients living in counties with high social vulnerability experienced the greatest pandemic-related decline.

The COVID-19 pandemic began in early 2020 and caused a substantial lockdown in the United States during March of that year.^29^ The pandemic placed an enormous burden on the health care system, leading to challenges that had a great impact on the ability to deliver health care services effectively.^41^ In particular, the pandemic had a significant impact on screening, causing a sharp decline in global screening rates for all cancers.^13,37,42^ The dramatic decrease in screening was undoubtedly due to cancellations of elective and planned procedures, as well as a decrease in public transport, loss of income to pay for services, and a shift in the health workforce toward COVID-19 services.^43,44^ To this point, Chen et al.^36^ reported that CRC screening rates were 13.1% lower in July 2020 than in July 2019. Similarly, Tinmouth et al.^45^ reported a 60% reduction in colonoscopies performed during 2020 versus 2019 in Canada. Other studies conducted in the Netherlands, Poland, United Kingdom, Israel, and Croatia also reported a decline in cancer screening in 2020 compared with pre-pandemic years.^46–49^ Furthermore, patients with a high SVI were more likely to be affected by COVID-19-related illnesses, which disproportionately affected their odds of being screened during the pandemic.^50^ In line with these studies, the current study noted a substantial decrease in median CRC screening volumes during 2020 after implementation of lockdown restrictions due to the COVID-19 pandemic. Interestingly, the post-pandemic screening rate did not rebound to pre-pandemic levels or surpass them. This may have been due to patient reluctance to seek medical care in the post-pandemic setting, as well as difficulty accessing cancer screening due to additional financial burdens related to the pandemic. Elderly patients and patients with comorbidities, who were at higher risk for serious complications from COVID-19, may have been specifically affected by these factors. Another reason for the smaller rebound in post-pandemic screening may have been the expansion of screening guidelines to include individuals 45 years old at a time when the physician workforce was constrained by pandemic-related challenges. Of particular note, individuals with moderate or high SVI had lower odds of screening before, during, and after the pandemic, with the worst odds reported for individuals residing in high-SVI areas during the pandemic. These findings highlight that socially vulnerable populations were most affected by pandemic-related policy changes.

Variations in cancer screening due to race/ethnicity, language, disability, and social demographics have been described.^16,43–46^ The COVID-19 pandemic exacerbated the obstacles faced by marginalized communities, resulting in restricted access to health care services such as cancer prevention and care.^41,51–53^ To this point, SVI may serve as a composite tool to assess key social determinants of health and provide insight into barriers for health care access and utilization. Bauer et al.^16^ reported that areas characterized by high SVI were associated with reduced screening rates. Moreover, individuals from high-SVI counties may not have access to effective transportation for travel to cancer screening facilities.^54,55^ Despite efforts to improve access to health care, patients who reside in moderate- and high-SVI areas still experience disparities in access to cancer prevention services.^16,56^ Building on previous work, the current study demonstrated that individuals in the high socially vulnerable areas experienced the lowest odds of CRC screening during the pandemic. In turn, these data serve to illustrate the ongoing need to address and bridge gaps in access to cancer prevention care.^57–59,60^ During 2021, numerous countries resumed their cancer screening programs and implemented strategies to boost screening rates.^61–63^ Additionally, efforts were made to ensure that individuals who missed their cancer screenings during to the pandemic were given priority and screened first.^62,63^ Resumption of these programs, complemented by the relaxation in pandemic lockdowns and increase in tele-medicine services, improved access to health care services globally.^64,65^ Interestingly, data from the current study demonstrated disparate access to CRC screening among various racial/ethnic groups. For example, Hispanic and other non-White racial/ethnic groups experienced a decline in the odds of CRC screening during the pandemic that did not recover to pre-pandemic levels. Barriers such as language difficulties, insurance status, limited health literacy, reduced awareness of preventive health care, and cultural differences may preclude certain racial/ethnic groups from accessing health services, which may worsen disparities in cancer prevention.^66^ Recently, health care organizations and policymakers have sought to improve access to care through initiation of the Affordable Care Act, as well as through outreach programs, mobile screening vans, and community-based awareness initiatives.^67,68^ In turn, these efforts may mitigate race/ethnicity-based disparities in cancer screening among non-Hispanic White and non-Hispanic Black individuals.^69,70^ In fact, non-Hispanic Black individuals may be more likely than non-Hispanic White individuals to be up to date relative to cancer screening.^71^

The current study may have important implications for health care institutions, payers, policymakers, and professional organizations. For example, the data offer evidence to support implementation of community policies and related initiatives focused on investment and promotion of structural equity within cancer prevention systems. Socially vulnerable patients from disadvantaged communities face additional barriers that preclude access to cancer care prevention. Therefore, focused efforts to coordinate health care delivery systems, screening for social determinants during clinical encounters, utilization of telehealth services, and home-based screening methods are needed to improve cancer care equity. The Colorectal Cancer Control Program (CRCCP), operated by the Centers for Disease Control and Prevention (CDC), offers financial support to states and Native American tribes with the aim of improving CRC screening rates among underserved groups.^72^ This initiative is focused on providing screening and follow-up services to individuals with low income and limited or no insurance coverage. The National Colorectal Cancer Roundtable (NCCRT) is a coalition of various organizations from the public, private, and voluntary sectors, that is also working to increase CRC screening rates across the country.^73^ Similar to CRCCP, their efforts primarily target low-income and underrepresented communities through initiatives such as public education campaigns, provider training, and policy advocacy. Health care providers and organizations must work together to improve screening of CRC patients, especially among vulnerable populations, to improve the quality of cancer prevention services.

The current study should be interpreted with consideration of several limitations. Because only Medicare beneficiaries were examined, the findings may not be generalizable to younger individuals.^74^ Although most screening methods are covered under Medicare, home testing kits using fecal occult blood tests may not fall under the coverage. Moreover, use of administrative data may be associated with data miscoding and misclassification errors. To address this, we used ICD-10-CM diagnosis codes previously demonstrated to have high fidelity for administrative data use.^75^ The Medicare data were available only until December 2021. As a result, the period after COVID-19 was not fully captured, leading to the utilization of monthly median screening volumes instead of overall screening rates. Moreover, the impact of the COVID-19 pandemic on different screening methods, including colonoscopy, fecal occult blood testing, and CT colonography, could not be assessed due to database limitations. The current study examined only CRC screening. Future studies need to assess the impact of the decrease in screening on the incidence of late-stage CRC and CRC mortality. The current study could not assess the percentage of individuals recommended for screening who were up to date with their screening according to USPSTF screening guidelines due to the limited study duration.

In conclusion, the COVID-19 pandemic caused a sharp decline in CRC screening after March 2020 that was followed by a recovery to near pre-pandemic levels after easing of restrictions in 2021. Individuals with high SVI and certain racial/ethnic groups experienced the greatest decline in screening volumes during the COVID pandemic. These data highlight the need to address social determinants of health to alleviate disparities in cancer care prevention.

### Supplementary Information

Below is the link to the electronic supplementary material.

Supplementary file1 (DOCX 343 KB)

## Data Availability

The data for this study were obtained from the Medicare Standard Analytic Files. There are restrictions to the availability of these data, which were used under license for this study. Data can be accessed with permission from the Centers for Medicare and Medicaid Services.
