# Supplementary material for: Association of COVID-19 Pandemic with Colorectal Cancer Screening: Impact of Race/Ethnicity and Social Vulnerability
Source: Ann Surg Oncol. 2024 Feb 15;31(5):3222–32. doi: 10.1245/s10434-024-15029-x (PMC10997707; doi:10.1245/s10434-024-15029-x)
Supplement: Supplementary file 1 — Supplementary file1 (DOCX 343 KB) [file 10434_2024_15029_MOESM1_ESM.docx]

**SUPPLEMENTARY APPENDIX
Association of COVID-19 Pandemic with Colorectal Cancer Screening Utilization in Relation to Social Vulnerability***Social Vulnerability Index (SVI)*

The SVI is a validated measure jointly developed and maintained by the Center for Disease Control and Agency for Toxic Substances and Disease Registry. It was employed to account for community vulnerability to external pressures. The county-level SVI was integrated with the Medicare Standard Analytical Files using county-level Federal Information Processing System (FIPS) codes and categorized into tertiles. The cut-off values were 44.3 and 71.5.^1^

- *Charlson Comorbidity Index (CCI)*

The CCI was used to evaluate comorbidity burden.^2^ (**Table 3**)

**Table 1.** Colorectal Cancer Screening Codes^3,4^

| **Cancer Screening** | **ICD 10 codes** | **CPT code** | **HCPC** | **ICD 9 codes** |
| --- | --- | --- | --- | --- |
| Colorectal | Z12.10 | 45378 (Colonoscopy) | G0327 | V76.51 |
| Colorectal | Z12.11 | 74263  (Computed tomographic colonography) | G0104 |  |
| Colorectal | Z12.12 | 81528  (Cologuard) | G0105 |  |
| Colorectal |  | 82270  (Fecal occult blood test) | G0121 |  |
| Colorectal |  | 82274 (Fecal Immunochemical Test) | G0106 |  |
| Colorectal |  |  | G0120 |  |
| Colorectal |  |  | G0107 |  |
| Colorectal |  |  | G0328 |  |
| Colorectal |  |  | G0122 |  |
| Colorectal |  |  | G0464 |  |

**Table 2.** Genetic Susceptibility ICD Codes^3^

|  | **ICD-10 Codes** |
| --- | --- |
| Genetic susceptibility to malignant neoplasms (CRC) | Z15.0 |
| Genetic susceptibility to other malignant neoplasm | Z15.09 |

**Table 3.** Charlson Comorbidity Index

| **Complication** | **Weight** | **ICD-9-CM** | **ICD-10-CM** |
| --- | --- | --- | --- |
| Acute myocardial infarction | 1 | 410.xx, 412.xx | I21.xx, I22.xx, I25.2 |
| Congestive heart failure | 1 | 428.xx | I50.xxx |
| Peripheral vascular disease | 1 | 441.xx, 443.9, 785.4, V434 | I71.xxx, I79.0, I73,9, R02, Z95.8xx, Z95.9 |
| Cerebral vascular accident | 1 | 430.xxx to 438.xxx | I60.xx, I61.xx, I62.xx, I63.xxx, I65.xx, I66.xx, G45.0, G45.1, G45.2, G45.8, G45.9, G46.xx, G45.4, I67.0, I67.1, I67.2, I67.4, I67.5, I67.6, I67.7, I67.8xx, I67.9, I68.0, I68.2, I68.8, I69.xxx |
| Dementia | 1 | 290.xx | F01.xxx, F02.xxx, F05 |
| Pulmonary disease | 1 | 490, 491.xx, 492.xx, 493.xx, 494.x, 495.x, 496, 500, 501, 502, 503, 504, 505 | J40, J41.x, J42, J43.x, J44.x, J45.xxx, J47.x, J67.x, J44.x, J60, J61, J62.x, J63.x, J64, J65, J66.x |
| Connective tissue disorder | 1 | 710.0, 710.1, 710.4, 714.0, 714.1, 714.2, 714.81, 725 | M32.xxx, M34.xx, M33.2xx, M05.3xxx, M05.8xxx, M05.9, M06.0xx, M06.3xx, M06.9, M05.0xx, M05.2xxx, M05.1xxx, M35.3 |
| Peptic ulcer | 1 | 531.xx, 532.xx, 533.xx, 534.xx | K25.x, K26.x, K27.x, K28.x |
| Diabetes | 1 | 250.0x, 250.1x, 250.2x, 250.3x, 250.7x | E10.9, E11.9, E13.9, E14.9, E10.1x, E11.1x, E13.1x, E14.1, E10.5x, E11.5x, E13.5x, E14.5 |
| Diabetes complications | 1 | 250.4x, 250.5x, 250.6x | E10.2x, E11.2x, E13.2x, E14.2x, E10.3xx, E11.3xx, E13.3xx, E14.3x, E10.4x, E11.4x, E13.4x, E14.4x |
| Paraplegia | 2 | 342.xx, 344.1 | G81.xx, G04.1, G82.0x, G82.1x, G82.2x |
| Renal disease | 2 | 582.xx, 583.0, 583.1, 583.2, 583.3, 583.5, 583.6, 583.7, 583.4, 585.x | N03.x, N05.2, N05.3, N05.4, N05.5, N05.6, N07.2, N07.3, N07.4, N01.x, N18.3x, N18.4, N18.5, N18.6, N19, N25.xx |
| HIV | 6 | 042, 043, 044 | B20, B21, B22, B23, B24 |

^ICD: International Classification of Diseases; HIV: human immunodeficiency virus.^

**Figure 1**. Figure showing new hospital admissions during COVID-19 pandemic by state/territory.^5^


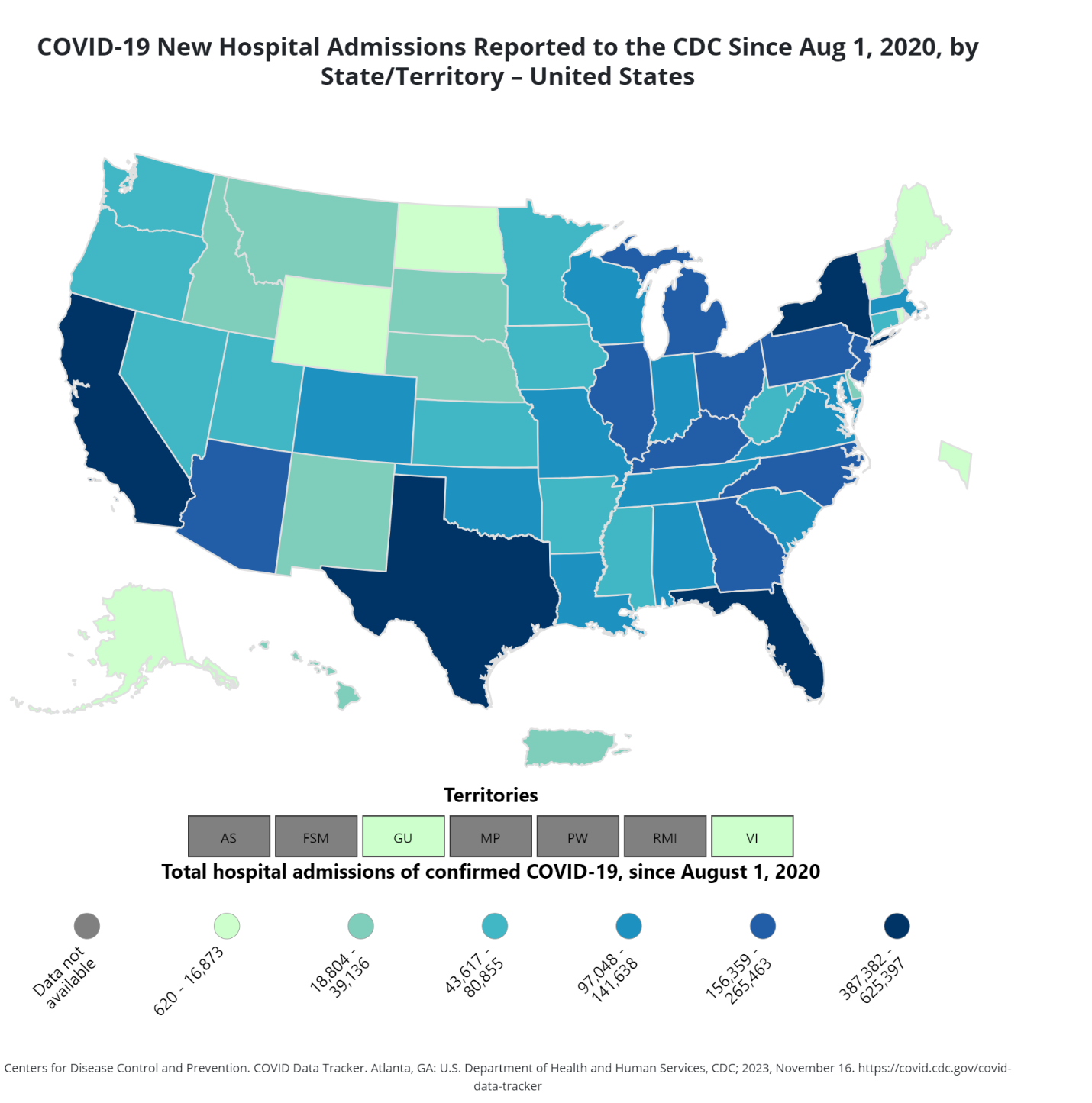


**References**

1. CDC/ATSDR Social Vulnerability Index (SVI). Published November 16, 2022. Accessed June 4, 2023. https://www.atsdr.cdc.gov/placeandhealth/svi/index.html

2. Charlson ME, Pompei P, Ales KL, MacKenzie CR. A new method of classifying prognostic comorbidity in longitudinal studies: development and validation. *J Chronic Dis*. 1987;40(5):373-383. doi:10.1016/0021-9681(87)90171-8

3. Free 2023 ICD-10-CM Codes. Accessed July 14, 2023. https://www.icd10data.com/ICD10CM/Codes

4. ICD - ICD-9-CM - International Classification of Diseases, Ninth Revision, Clinical Modification. Published November 3, 2021. Accessed June 8, 2023. https://www.cdc.gov/nchs/icd/icd9cm.htm

5. CDC. COVID Data Tracker. Centers for Disease Control and Prevention. Published March 28, 2020. Accessed November 16, 2023. https://covid.cdc.gov/covid-data-tracker
